# Ultrastructure of Plant Leaf Cuticles in relation to Sample Preparation as Observed by Transmission Electron Microscopy

**DOI:** 10.1155/2014/963921

**Published:** 2014-04-22

**Authors:** Paula Guzmán, Victoria Fernández, Mohamed Khayet, María Luisa García, Agustín Fernández, Luis Gil

**Affiliations:** ^1^Forest Genetics and Ecophysiology Research Group, School of Forest Engineering, Technical University of Madrid, Ciudad Universitaria s/n, 28040 Madrid, Spain; ^2^Department of Applied Physics I, Faculty of Physics, University Complutense of Madrid, Avenida Complutense s/n, 28040 Madrid, Spain; ^3^Electron Microscopy National Centre, Faculty of Chemistry, University Complutense of Madrid, Avenida Complutense s/n, 28040 Madrid, Spain

## Abstract

The leaf cuticular ultrastructure of some plant species has been examined by transmission electron microscopy (TEM) in only few studies. Attending to the different cuticle layers and inner structure, plant cuticles have been grouped into six general morphological types. With the aim of critically examining the effect of cuticle isolation and preparation for TEM analysis on cuticular ultrastructure, adaxial leaf cuticles of blue-gum eucalypt, grey poplar, and European pear were assessed, following a membrane science approach. The embedding and staining protocols affected the ultrastructure of the cuticles analysed. The solubility parameter, surface tension, and contact angles with water of pure Spurr's and LR-White resins were within a similar range. Differences were however estimated for resin : solvent mixtures, since Spurr's resin is combined with acetone and LR-White resin is mixed with ethanol. Given the composite hydrophilic and lipophilic nature of plant cuticles, the particular TEM tissue embedding and staining procedures employed may affect sample ultrastructure and the interpretation of the results in physicochemical and biological terms. It is concluded that tissue preparation procedures may be optimised to facilitate the observation of the micro- and nanostructure of cuticular layers and components with different degrees of polarity and hydrophobicity.

## 1. Introduction

The epidermal cells of most aerial plant organs including leaves, flowers, fruits, and nonwoody stems are covered with an extracellular membrane named cuticle, which plays a crucial physiological role [1] and provides protection against multiple potential biotic and abiotic stress factors [2]. The high degree of variation in cuticle thickness, structure, and chemical composition among plant species and varieties, organs, states of development, and environmental stress conditions during growth [3, 4] reflects the heterogeneous and composite nature of this membrane. For example, the cuticular ultrastructure of the seagrass* Halodule wrightii* was observed to be modified when in contact with the epiphytic alga* Hincksia mitchelliae *[5].

As a major chemical constituent, the cuticle is normally composed of a cutin and/or cutan biopolymer matrix, waxes both intruded into (intracuticular) and deposited on to (epicuticular) the matrix, polysaccharides, and phenolics [6].

According to the model of von Mohl [7], the cuticle consists of two different layers, that is, the cuticle proper (CP) as the outermost zone and the cuticular layer (CL), which is located in between the CP and the outer cell wall. In cuticles from several species, the CL can be divided into an external cuticular layer (ECL) and an internal cuticular layer (ICL), which correspond to the outer and inner zones of the CL, respectively [8]. Additionally, an epicuticular wax (EW) layer covers the CP and is in direct contact with the surrounding atmosphere. The CP has been believed to be composed of waxes and cutin/cutan, while polysaccharide material is additionally present in the CL [8]. Nevertheless, Guzmán et al. [9] provided evidence for the presence of polysaccharides in different cuticle areas of poplar, eucalypt, and pear leaves, including also the CP. At least six different types of ultrastructures have been identified in plant cuticle transversal sections and the different layers have been generally described as either amorphous, lamellate, or reticulate [8, 10].

Transmission electron microscopy is a useful tool for examining the ultrastructure of biological materials (e.g., [11, 12]), which has been applied in plant cuticle anatomical studies [13]. The majority of these investigations were performed during the 1980s with few plant species and organs (e.g., [10, 14]). The state of scientific knowledge is such that it is currently not possible to establish a link between cuticle chemical composition and ultrastructure [15]. Furthermore, the preparation of tissues for TEM observation involves procedures that could chemically interact with their components and hence alter the natural arrangement and appearance of such tissues.

The permeability of a compound through a plant cuticle is the product of its solubility, which is a thermodynamic parameter reflecting the degree of interactions between a compound and the plant cuticle, and its diffusivity through the cuticle, which is a kinetic parameter associated with the size of the compound and the structure of the matrix. Therefore, the solubility between cuticular constituents and TEM tissue preparation chemicals (e.g., solvents, resins, or stains) together with their diffusivity in the cuticle matrix (largely influenced by the surface tension of the different solvents, resins, and resin mixtures employed and also by their molecular size) may influence the quality and interpretation of cuticle TEM micrographs.

Prediction of solubility parameters is commonly used, for example, in the design and fabrication of polymeric membranes [16, 17], in the coating industry [18], and also in pharmacology [19]. The total solubility parameter and solubility parameter components (i.e., the apolar, polar, and hydrogen- (H-) bonding components) of model plant surface chemical constituents have been recently estimated by Khayet and Fernández [15].

This study was aimed at analyzing the leaf cuticular ultrastructure of three model plant species as affected by different sample preparation methods. Leaves and cuticles embedded in Spurr's and LR-White resin were prepared according to standard TEM procedures. Spurr's resin was selected since it is frequently used for observation of plant cuticles by TEM [14]. LR-White resin was utilised for comparison, since it is often used for analysing biological materials especially in immunohistochemical and histological studies [9, 20], it involves less tissue handling steps, and such resin is considered to be more hydrophilic [21, 22]. The European pear leaf was selected since it has been previously described [23] and has a cuticular structure which does not fit within the existing six cuticular types suggested by Holloway [8, 10]. The leaf cuticle of grey poplar has been analysed since it has been used as a model for the development of cuticular permeability studies [24]. The blue-gum eucalypt leaf has been examined due to its evergreen nature and markedly different ecophysiological habitat as compared to the other two tree species [9].

## 2. Material and Methods

### 2.1. Plant Material

Leaves of blue-gum eucalypt (*Eucalyptus globulus* Labill.), grey poplar (*Populus* x* canescens *(Ait.) Sm.), and European pear (*Pyrus communis* L. var. Blanca de Aranjuez) were selected for experimental purposes. Juvenile blue-gum eucalypt leaves were collected from 1.5-year-old seedlings growing in the Forest Engineering School Arboretum (Technical University of Madrid, Spain). Grey poplar leaves were obtained from trees genotyped with nuclear microsatellite markers grown in Losana (Soria, Spain; Sierra, Personal communication). European pear leaves were collected from trees grown in the Royal Botanic Gardens of Madrid (CSIC, Spain). Fully expanded, undamaged leaves from medium size shoots were collected during the summer. For simplicity, these species will be referred to as eucalypt, poplar, and pear throughout the paper. Leaves were collected for cuticle isolation (only analysing the adaxial leaf side) and also to be directly examined as intact tissues. Prior to isolating the cuticles enzymatically [25], leaf midveins and margins were removed with a scalpel. The enzymatic solution contained 2% cellulase, 2% pectinase (both from Novozymes, Bagsvared, Denmark) plus 1% polyvinylpyrrolidone (Sigma-Aldrich, Munich, Germany), and 2 mM sodium azide. The pH was adjusted to 5.0 by adding sodium citrate. Cuticles were maintained in solution (changed after two weeks) until they appeared to be fully separated from the underlying tissues. This took one month in the case of eucalypt and pear leaves and from one and a half to two months in the case of poplar. Leaf tissues were digested at room temperature (23 to 25°C) and solutions were manually shaken at frequent time intervals. After the extraction period, clean intact adaxial cuticles were selected, thoroughly washed in deionized water, air-dried, and stored for microscopic examination.

### 2.2. Tissue Fixation and Embedding

Isolated cuticles and fresh leaves were cut into approximately 4 mm^2^ pieces with a scalpel and subsequently subjected to different fixation and embedding protocols as follows.

#### 2.2.1. Spurr's Resin Embedding

Samples were fixed in 2.5% glutaraldehyde-4% paraformaldehyde (both from Electron Microscopy Sciences (EMS), Hatfield, USA) for 6 h at 4°C, rinsed in ice-cold phosphate buffer, pH 7.2, four times within a period of 6 h, and left overnight. Tissues were then postfixed in a 1 : 1 aqueous solution of 2% osmium tetroxide (TAAB Laboratories, Berkshire, UK) and 3% potassium ferrocyanide (Sigma-Aldrich) for 1.5 h. Samples were then washed with distilled water (x3), dehydrated in a series of 30, 50, 70, 80, 90, 95, and 100% acetone (x2, 15 min each concentration), and embedded in acetone-Spurr's resin (TAAB Laboratories) mixtures (3 : 1, 2 h; 1 : 1; 2 h; 1 : 3; 3 h (v : v)) and in pure resin overnight at room temperature. Samples were finally embedded in blocks which were incubated at 70°C for 3 days until complete polymerization.

#### 2.2.2. LR-White Resin Embedding

Samples were fixed in 2.5% glutaraldehyde-4% paraformaldehyde for 4 h at 4°C and washed in phosphate buffer as described above. They were then dehydrated in an ethanol series of 30, 50, 70, 80, 90, 95, and 100% (x2, 15 min each concentration) and embedded in ethanol-LR-White resin (London Resin Company, London, UK) mixtures (3 : 1, 1 h; 1 : 1; 1 h; 1 : 3; 2 h (v : v)) and in pure resin overnight. This embedding protocol was performed on ice. Thereafter, plant tissues were embedded in capsules which were subsequently incubated at 50°C for 2 days.

### 2.3. Estimation of Physicochemical Properties of the Resins

Advancing contact angles of water with resin films and the surface tension of Spurr's and LR-White resins were determined using a CAM 200 contact angle meter (KSV Instruments, Helsinki, Finland) equipped with a CCD camera, frame grabber, and image analysis software. Pure resins were smeared onto microscope slides and polymerized under the same conditions used for embedding plant tissues. Flat, approximately 2 mm thick films were obtained after resin polymerization. Advancing contact angles of 2 *μ*L drops of double-distilled water were measured at room temperature using manual dosing system holding a 1 mL syringe with 0.5 mm diameter needle (10 repetitions). The surface tension of the resins was determined by the pendant drop method, using a 1 mL syringe with 1.8 mm diameter needle (15 repetitions). Side view images of the drops were captured at a rate of 6 frames s^−1^. Contact angles and surface tensions were automatically calculated by fitting the captured drop shape to the one calculated from the Young-Laplace equation. The approximate solubility parameters of Spurr's [26] and LR-White (London Resin Company product datasheet) resins were predicted based on the method of van Krevelen and Hoftyzer [27], as described by Khayet and Fernández [15]. The molecular structures and molar volumes of resin constituents and solvents were obtained from ChemSpider (Royal Society of Chemistry, UK). The solubility parameters of the resin mixtures were calculated considering the relative proportion of chemical constituents of each resin type [27].

### 2.4. Transmission Electron Microscopy

Ultrathin tissue sections were mounted on nickel grids and observed with a Jeol 1010 electron microscope (Tokyo, Japan) equipped with a CCD Megaview camera, operated at 100 kV. Prior to TEM observation, sections were poststained with 2% aqueous uranyl acetate for 20 min (LR-White embedded samples) and Reynolds' lead citrate (both chemicals from EMS) for 5 min (Spurr's and LR-White embedded samples).

### 2.5. Scanning Electron Microscopy

Gold-sputtered intact eucalypt, poplar, and pear adaxial leaf surfaces and enzymatically digested adaxial leaf cuticles of the same species were examined with a Hitachi S-3400 N (Tokyo, Japan) scanning electron microscope (SEM).

## 3. Results

### 3.1. Cuticle Isolation Procedure

**Figure 1:**
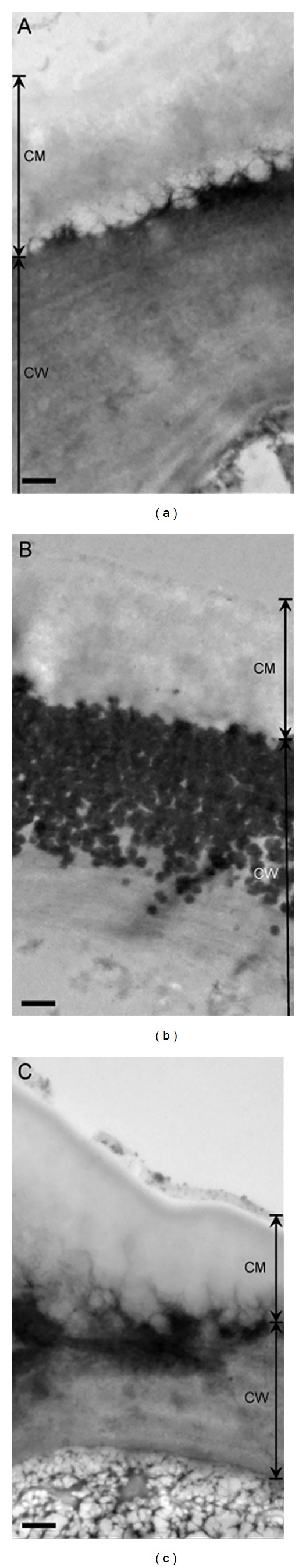
Transversal sections of the adaxial cuticular membrane (CM) and outer cell wall (CW) of intact leaves of eucalypt (a), poplar (b), and pear (c) embedded in LR-White resin. Bars: 500 nm.

The enzymatic digestion process enabled the isolation of intact, unbroken cuticles of eucalypt and pear leaves. However, only small poplar cuticle pieces could be recovered, which were always attached at least to the outer epidermal cell wall (containing dark, granular structures as observed in Figure 1(b)).

**Figure 2:**
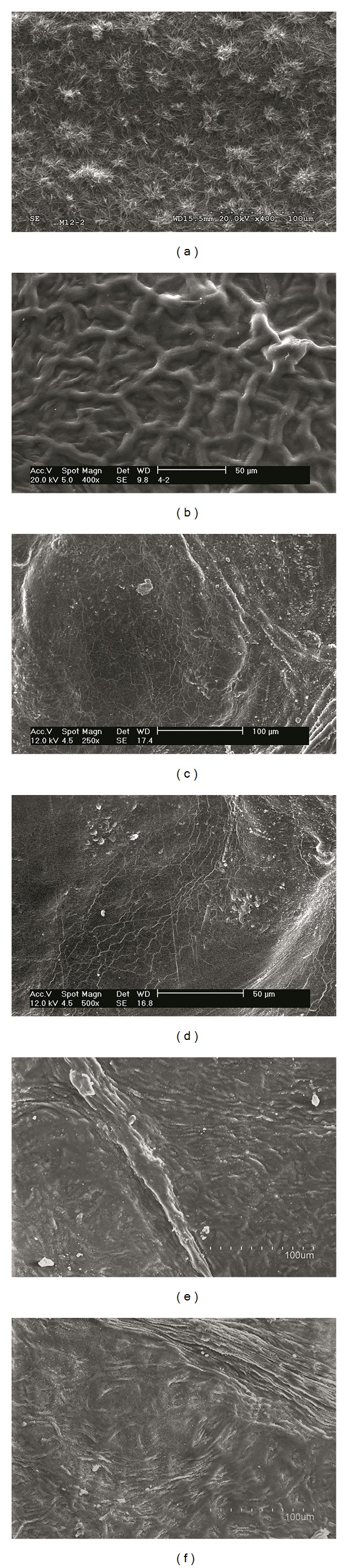
Influence of enzymatic digestion on the outer surface of eucalypt, poplar, and pear adaxial cuticles (b, d, and f) as compared to intact leaves (a, c, and e). (a, b): x500; (c–f): x400.

After SEM observation of the surface of intact leaves versus enzymatically digested cuticles (Figure 2), we noticed a major change concerning the topography of the eucalypt surface. The enzymatic isolation process washed off the wax nanotubes covering the leaf surface (Figure 2(a)), leading to a smoother cuticle topography, which revealed the shape of underlying epidermal cells (Figure 2(b)). In contrast, no remarkable changes were observed when comparing the topography of the outer surface of poplar and pear leaves and isolated cuticles (Figures 2(c)–2(f)).

### 3.2. Sample Preparation Procedure and Cuticular Ultrastructure

**Figure 3:**
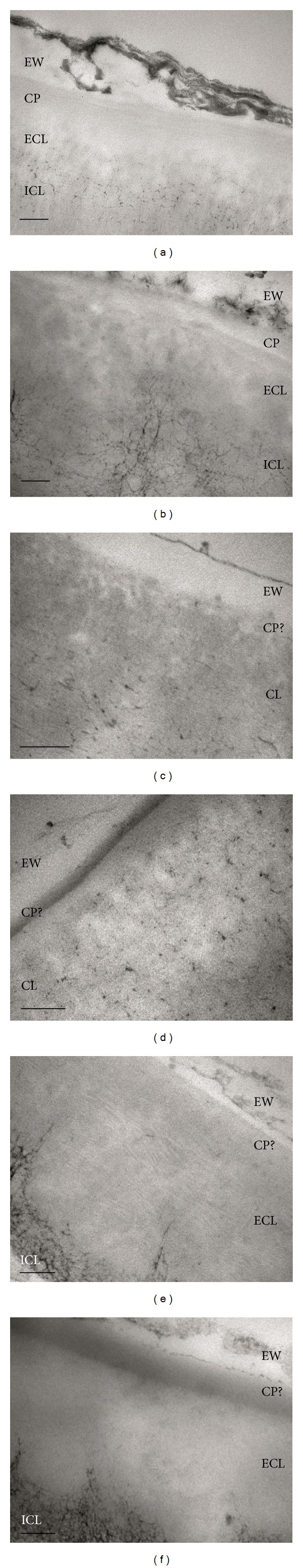
Ultrastructure and cuticular layers of eucalypt, poplar, and pear cuticles embedded in Spurr's (a, c, and e) and LR-White resin (b, d, and f). EW: epicuticular waxes, CP: cuticle proper, CL: cuticular layer, ECL: external cuticular layer, and ICL: internal cuticular layer. Bars: 200 nm.

For the same species, differences in the leaf cuticle ultrastructure were identified in relation to the TEM procedures analysed. A similar ultrastructure was observed for the eucalypt cuticle when embedded in both resin types. The ICL, ECL, and CP can be defined as reticulate, amorphous, and faintly lamellate, respectively (Figures 3(a) and 3(b)). However, the CP lamellae appeared more uniform and conspicuous in Spurr's embedded cuticles (Figure 3(a)). A thin, electron-dense band was observed just below the EW layer in LR-White embedded cuticles (Figure 3(b)). The poplar CL was observed to be reticulate in cuticles embedded in both resin types, with the ICL and ECL being not distinguishable (Figures 3(c) and 3(d)). In Spurr's resin embedded cuticles, the CP could not be differentiated from the CL (Figure 3(c)), while some micrographs of LR-White resin embedded cuticles showed an amorphous or slightly reticulate, electron-dense band, which may correspond to this layer (Figure 3(d)). The pear cuticle ultrastructure had a different appearance when embedded in the two resins. While the ICL was always reticulate (Figures 3(e) and 3(f)), the ECL can be described as lamellate or amorphous when using Spurr's (Figure 3(e)) or LR-White resin (Figure 3(f)), respectively. The orientation of the lamellae was observed to be mainly parallel to the outer epidermal cell wall (Figure 3(e)). The CP may be represented by a more external, lamella-free band sometimes appearing slightly electron-lucent (Figure 3(e)) or electron-dense (Figure 3(f)) in Spurr's or LR-White resin embedded cuticles. A very thin, discontinuous, electron-dense band, in contact with the EW layer and similar to that observed in LR-White resin embedded eucalypt cuticle, could also be detected when using Spurr's resin (Figure 3(e)).

A dark and apparently amorphous layer stemming from the middle lamella (i.e., likely pectinaceous) into the cuticle was chiefly observed in pear (Figure 1(c)) and also in eucalypt (Figure 1(a)). However, a granular layer was noticed in poplar leaves (Figure 1(b)) and cuticles (data not shown [9]) in a similar location.

### 3.3. Resin Properties

Some physicochemical properties of the resins, which may influence tissue embedding, were estimated. The contact angles of water with resin films were (°; mean ± standard deviation) 46.6 ± 2.7 and 28.7 ± 2.2 for Spurr's and LR-White resins, respectively. The surface tension (mN m^−1^; mean ± standard deviation) was 32.64 ± 0.23 and 32.56 ± 0.21 for LR-White and Spurr's resins. The total solubility parameters**δ** of Spurr's and LR-White resins were similar and ranged between 19.0 and 18.7 MJ^1/2^ m^−3/2^ (Table 1).

When mixing resins with organic solvents, the total **δ**  of the mixtures hardly varied regarding Spurr's: acetone while for increasing LR-White: ethanol ratios (from 1 : 3 to 3 : 1) it ranged between 25 and 21 MJ^1/2^ m^−3/2^. The total **δ**  value of common model cuticular components [15] is shown in Table 2. The affinity between cuticular components and the chemicals employed for tissue handling can be estimated by their total **δ**  increment (Δ**δ** [15]). Embedding media of low LR-White: ethanol ratios will have a higher affinity for polysaccharides, while pure LR-White and Spurr's based media will have a greater affinity for waxes and cutin.

## 4. Discussion

In this study, the ultrastructure of the adaxial leaf cuticle of three tree species was analysed by TEM. The cuticle transversal sections of eucalypt, poplar, and pear had different inner structures and cuticular layers, which were differentially affected by the TEM tissue preparation procedures analysed.

### 4.1. Cuticle Isolation and Attachment to the Underlying Cell Wall

Concerning the preliminary process of enzymatic cuticle isolation, large pieces could be extracted from eucalypt and pear leaves in contrast to poplar cuticles, which broke into small pieces. Eucalypt and especially pear leaf cuticles have been successfully isolated in several studies (e.g., [9, 23, 29]). The adaxial cuticle of grey poplar leaves has been used by Schönherr and coworkers as a model for the development of permeability studies (e.g., [20, 30]). Examination of cross-sections of enzymatically digested grey poplar leaf cuticles by TEM revealed that they were attached at least to the outer layer of the epidermal cell wall. If we consider the cuticle as the region located above the cell wall as defined by Norris and Bukovac [23], it can be concluded that grey poplar cuticles (the species of the individuals analysed was confirmed with nuclear microsatellite markers) cannot be isolated via cellulase and pectinase digestion, which may raise questions on the potential species or hybrids used in previous cuticular permeability studies [20, 30]. On the other hand, the severe loss of EW of eucalypt leaf cuticles during the enzymatic isolation process (Figures 2(a) and 2(b)) suggests that investigations developed with isolated cuticles should be interpreted with caution, since they may lead to significant chemicals and/or structural modifications in the extracted tissues. Digestion of the pectin-rich layer extending into the cuticle of eucalypt and pear may facilitate the separation of the tissues along this zone, as previously suggested [23, 25]. In contrast, the granular layer observed in the outer cell wall of poplar [9] could not be hydrolysed by cellulase and pectinase. Therefore, the occurrence of structural and chemical cell wall and cuticular variations in relation to different species may ease or impede the process of cuticle enzymatic isolation, but the existing relationship remains unclear.

### 4.2. Effect of Sample Preparation on Cuticular Ultrastructure

Differences in the cuticular ultrastructure of the same species were detected in relation to the TEM procedures employed. The permeability of a compound through a plant cuticle is the product of its solubility (a thermodynamic parameter) and its diffusivity through the cuticle matrix (a kinetic parameter) [15]. It can be expected that fixation, embedding, and staining media must permeate the cuticle as a prerequisite for tissue preparation for TEM. For simplicity and in order to hypothesise the mechanisms involved, we will assume that cuticle structure and composition will keep moderately unchanged during the sample preparation process, as observed when comparing intact leaf and isolated cuticle TEM micrographs (Figures 2 and 3). Considering this assumption, the major factor affecting sample ultrastructure would be the exiting solubility (affinity) between cuticular components and TEM tissue preparation chemicals.

Tissues were embedded in Spurr's and LR-White resins and were dissolved in acetone and ethanol, respectively. The latter resin is generally considered hydrophilic, in contrast to the Spurr's resin, which is believed to be more hydrophobic (e.g., [22, 31]). However, in this study we found no major differences between the solubility parameter and the surface tension of pure Spurr's and LR-White embedding media. According to the low contact angles of pure resin films with water (about 47° for Spurr's resin and 29° for LR-White resin), both polymer mixtures can be considered as wettable (contact angles with water below 90°). Furthermore, differences were estimated for the total**δ**  of resin: solvent mixtures, with LR-White media having higher values (from 25 to 21 MJ^1/2^ m^−3/2^ for increasing ratios of resin : ethanol) as compared to Spurr's resin (from 20 to 19 MJ^1/2^ m^−3/2^ for increasing ratios of resin : acetone). Additionally, the lower viscosity of LR-White may somehow facilitate tissue infiltration as compared to Spurr's resin.

A low solubility parameter difference (Δ**δ**) between two chemicals indicates a higher affinity between them [15]. In light of the estimated total **δ**, both resins will theoretically have a higher affinity for cutin and waxes and a lower affinity for polysaccharides. LR-White: ethanol mixtures are however more polar (chiefly due to the higher H-bonding component of the solvent) and will consequently have a higher affinity for polysaccharides than pure LR-White resin and Spurr's resin mixtures, especially for the highest ethanol concentration.

The composite nature of the cuticle in terms of hydrophilic and lipophilic constituents should be regarded when attempting to interpret cuticular permeability to embedding, fixation, and staining media. Since all the TEM dyes are dissolved in distilled water, which has a high surface tension (72 mN m^−1^ at 25°C) and a high total **δ** (47.9 MJ^1/2^ m^−3/2^) [28], it can be assumed that they may chiefly permeate the cuticle via the hydrophilic pathway [32]. It must be noted that plant tissues were infiltrated at atmospheric pressure with resins, acetone or ethanol mixtures, and stain aqueous solutions. Under such conditions it is likely that small diameter, cuticular nanopores [32] may not be easily infiltrated by the different liquids. Thereby, the polar domains located in the more superficial areas of the cuticle and leaf sections will be stained more easily, and their structure will be better identified by TEM. Similarly, the cuticle zones preferentially enabling the infiltration of resin: solvent mixtures and/or pure resin as a result of their structure and/or chemical composition will be better preserved and contrasted with the dyes.

Following the common cuticular structure terminology [8, 10] and as major differences, the ECL of pear was observed to be lamellate and amorphous when embedded in Spurr's and LR-White resins, respectively. The mainly parallel orientation to the outer epidermal cell wall of the lamellae differs from the chiefly perpendicular one observed in Holloway's micrographs [10]. Poplar cuticles embedded in Spurr's resin showed either a reticulate CP, which could not be distinguished from the CL underneath, or an absence of such layer. In contrast, a discontinuous, electron-dense band was observed in some micrographs of LR-White embedded samples, which could be considered as amorphous or reticulate, depending on the cuticle portion examined. The pear CP may be interpreted as a slightly electron-lucent band sometimes observed in Spurr's embedded cuticles, which appeared to be electron-dense in LR-White embedded cuticles.

Holloway's ultrastructural types are often used as tools for analysing plant cuticles [8, 10]. However, a fair degree of variation and different interpretations may derive from the particular sample preparation procedure. Thus, the eucalypt leaf cuticle embedded in both resins could be assigned to Holloway's Type 2, the poplar leaf cuticle to Type 4 if embedded in Spurr's resin and to Type 3 or 4 if embedded in LR-White resin (depending on the micrograph), and finally the pear leaf cuticle to Type 3 [10, 33] after embedding in LR-White resin and to Type 7, as suggested by Jeffree [8] after embedding in Spurr's resin. It is likely that the structure of the most polar cuticular regions, which may also be associated with a lower degree of impregnation with lipid compounds, is better preserved after tissue infiltration in LR-White media, but the influence of tissue staining should also be considered. Thereby, it can be concluded that the whole sample preparation protocol may lead to ultrastructural variations as reported in this investigation. Owing to the composite chemical nature of the plant cuticle, the methods may be adjusted to enable optimal observation of different cuticular layers and/or chemical constituents [9]. Furthermore, we showed the occurrence of ultrastructural variations in relation to different TEM sample preparation methods, which should be taken into account when attempting to establish a general morphological classification of plant cuticles.

## 5. Conclusion

The ultrastructural observation of the leaf cuticle of three plant species processed for TEM by different methods suggests that classification of cuticular ultrastructures according to specific and oversimplified patterns must be interpreted with caution. The observed structural variations between the same or among different species may arise from multiple factors, such as, for example, the tissue embedding protocol, the resin polymerization temperature, the TEM staining process, the observed cuticle area, or plant phenological or environmental changes during growth and development. Moreover, since samples are embedded and stained at ambient pressure and cuticles may contain nanoscale pores [32], it is likely that tissues may not be easily infiltrated by the resins and dyes. In addition, the low specificity of the stains limits our understanding of cuticular ultrastructure in relation to chemical composition [34]. Given the chemical and physical heterogeneity of plant cuticles [15], it can be expected that the degree of infiltration and staining will vary according to the solubility and diffusivity in the cuticle of all the solutes and solvents used during sample processing for TEM analysis. For instance, polysaccharide structure could be better observed in LR-White embedded tissues while lipidic cuticular components were better identified in Spurr's resin embedded samples. Therefore, the current staining and embedding protocols may be further improved and modified for better preserving the micro- and nanostructure of cuticular layers and/or chemical constituents with different degrees of polarity and hydrophobicity and according to the particular aims of each specific investigation.

## Figures and Tables

**Table 1:** Total solubility parameter (*δ*) and solubility parameter components (*δ*_*d*_, *δ*_*p*_, *δ*_*h*_) of Spurr's and LR-White resin chemical components.

Chemicals	Solubility parameter components (MJ ^1/2 ^ m ^−3/2^)			Total *δ*
	*δ*_*d*_	*δ*_*p*_	*δ*_*h*_	(MJ ^1/2^ m ^−3/2^)
*Spurr's resin *				
Nonenyl succinic anhydride	16.7	7.7	6.2	19.4
4-Vinyl-1-cyclohexene diepoxide	14.0	7.0	7.3	17.3
Propylene glycol diglycidyl ether	14.9	9.7	8.5	19.7
Dimethylaminoethanol	14.6	16.5	10.5	24.5
*Pure Spurr's resina *	—	—	—	19.0
Acetone^c^	15.5	10.4	6.9	19.9
1 Resin : 3 Acetone	—	—	—	19.7
1 Resin : 1 Acetone	—	—	—	19.4
3 Resin : 1 Acetone	—	—	—	19.2

*LR-White resin *				
2,2-Propanediylbis(4,1-phenyleneoxy-2,1ethanediyl) bis(2-methylacrylate)	16.7	3.1	7.0	18.3
2-Propenoic acid, 2-methyl-, dodecyl ester	19.1	1.7	4.9	19.8
*Pure LR-White resinb *	—	—	—	18.7
Ethanol^c^	15.8	8.8	19.4	26.4
1 Resin : 3 Ethanol	—	—	—	24.5
1 Resin : 1 Ethanol	—	—	—	22.5
3 Resin : 1 Ethanol	—	—	—	20.6

^a^26.0 g nonenyl succinic anhydride, 10.0 g 4-vinyl-1-cyclohexene diepoxide, 6.0 g propylene glycol diglycidyl ether, and 0.4 g dimethylaminoethanol [22].

^b^Assuming a concentration of 75% 2,2-propanediylbis(4,1-phenyleneoxy-2,1-ethanediyl), bis(2-methylacrylate) and 25% 2-propenoic acid, 2-methyl-, and dodecyl ester.

^c^Senichev and tereshatov [28].

**Table 2:** Total solubility parameter (*δ*) of common model cuticular compounds [15].

Chemicals	Total *δ* (MJ ^1/2^ ** **m^ −3/2^)
Waxes	17
Cutin monomers^a^	17–20
Polysaccharides^b^	31–33

^a^Assuming the occurrence of ester bonds.

^b^Assuming the occurrence of two glycosidic bonds.
